# Rare Non-obstetric Large Vulvar Hematoma Secondary to Consensual Sexual Intercourse: A Trial of Conservative Management

**DOI:** 10.7759/cureus.52850

**Published:** 2024-01-24

**Authors:** Lua Saylany, Hasthika Ellepola

**Affiliations:** 1 Obstetrics and Gynaecology, Logan Hospital, Logan, AUS

**Keywords:** vulva, non-obstetric traumatic vulvar hematoma, evacuation of hematoma, surgical intervention, vulvar trauma, vulvar hematoma

## Abstract

Outside of childbirth, a vulvar hematoma is an uncommon gynecological presentation that typically occurs secondary to blunt trauma to the perineum. Given the low incidence of non-obstetric vulvar hematoma, there is no standard guideline or consensus for managing such cases. There are two mainstay approaches: conservative management and surgical intervention. We present a case of a large vulvar hematoma secondary to forceful consensual sexual intercourse with a trial of conservative management followed by surgical intervention.

## Introduction

The vulvar region has a rich vascular supply through the internal pudendal artery, which can be easily compromised by blunt impact against the osseous structures of the pelvis, leading to the formation of vulvar hematoma [1]. Vulvar hematomas most commonly form in an obstetric setting (i.e., post a vaginal birth), either through direct injury (e.g., instrumental delivery, episiotomy, and/or perineal tears) or indirect injury (e.g., birth canal stretching) [2]. Non-obstetric vulvar hematomas are less common. Single case reports of non-obstetric vulvar hematomas have been reported in various settings, including bicycle accidents, falls onto firm surfaces, sexual assault, insertion of foreign objects into the vagina, and after vulvar surgery [3-6]. 

While most non-obstetric vulvar hematomas can be controlled and managed conservatively, significant complications may arise. It is important to consider that vulvar hematomas can rapidly grow in size, causing haemodynamic instability, can become infected if there is sub-optimal skin integrity and/or laceration of the genital tract, and can be incapacitating due to discomfort [2-5]. 

Initial patient assessment should involve a detailed history and physical examination. The history should include the mode of trauma, effect on mobility, assessment of neurological and urological symptoms, and qualification of pain. The physical examination should include an assessment of the hematoma size and extension to surrounding structures, including the urethra and pelvic floor [2,5]. 

As there are few cases of non-obstetric vulvar hematomas reported in the literature, there are no guidelines for management. Data needs to be established and evaluated to determine whether a patient needs conservative versus surgical management based on their clinical presentation. 

We present a case of a large vulvar hematoma secondary to forceful consensual sexual intercourse with a trial of conservative management. 

## Case presentation

**Figure 1:**
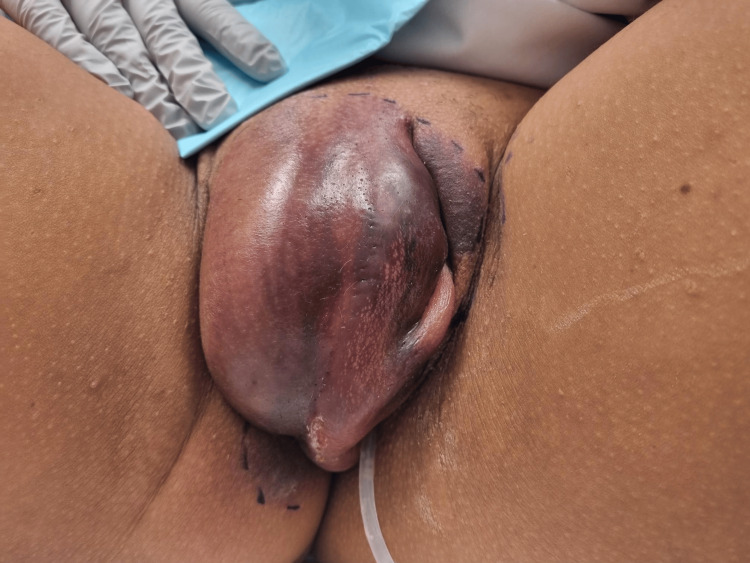
Large right-sided hematoma of the vulva with overlying ecchymosis extending from the right labia majora, the clitoris, and to the superior aspect of the left labia majora; indwelling urethral catheter in situ

A G2P2 28-year-old female presented to the emergency department reporting increasing right-sided perineal pain and swelling over a 24-hour period following an episode of consensual intercourse involving repeated insertion of a fist into the vagina by a casual sexual partner. She had no significant medical conditions and was not taking any medications. The patient was confidentially counselled regarding sexual assault and substance use. She stated that the sexual intercourse was consensual and that no substances were used. She had two previous uncomplicated spontaneous vaginal births with no known significant perineal trauma. She presented to the emergency department as the pain and swelling had limited her mobility, and she required assistance from a four-wheel walker to mobilize. She was referred to, and immediately seen by, the gynecology team. Her vital signs were stable, and her initial neurological examination was within normal limits. Assessment of the external genitalia revealed a large hematoma of the right vulva measuring 10 cm by 6 cm with overlying ecchymosis. The area was tender to light palpation and almost completely obstructed the urethral opening. An indwelling urethral catheter was inserted on initial examination to prevent urinary obstruction. She did not tolerate a speculum examination; however, she was able to tolerate a bimanual examination. There was no evidence of bleeding or laceration of the genital tract, and the pelvic floor muscles and vaginal wall were normal, with no evidence of extension to these surrounding structures. A surgical marker was used to outline the border of the hematoma and ecchymosis for surveillance (Figure 1). Her white cell count, c-reactive protein level, hemoglobin and platelet levels were all within normal limits. An urgent CT angiogram of the pelvis was performed, which did not show any evidence of arterial bleeding or injury to the pelvic structures; it only demonstrated a large localized right vulvar hematoma. 

The patient reassuringly remained hemodynamically stable with no neurological or urological signs or symptoms. The hematoma was stable in size after one hour of observation, and her pain was being controlled well with simple analgesia. Given this relative stability, the gynecology team discussed conservative versus surgical management options with the patient. The patient decided to try conservative management. This included commencing prophylactic intravenous antibiotics (cefazolin and metronidazole), regular and, as needed, analgesia, and regular ice packs applied to the perineum as tolerated. The anesthetic team was informed of the patient's admission in the event that she became hemodynamically unstable and required emergency surgical intervention. 

**Figure 2:**
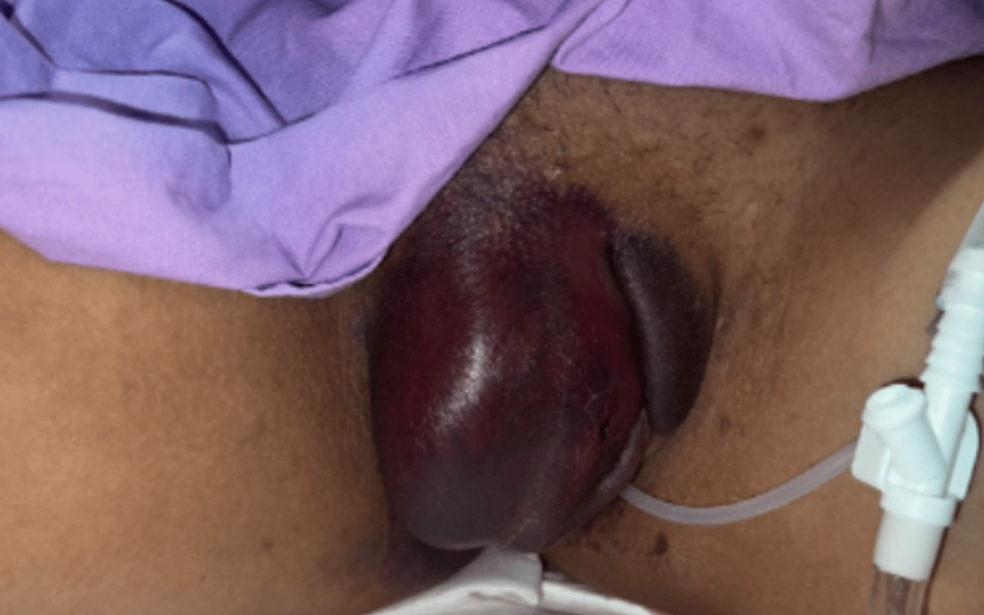
Approximately 72 hours after a trial of conservative management; indwelling urethral catheter in situ

Over the 72 hours of conservative management in the hospital, the patient's pain had gradually increased, and there was a significant spread of the ecchymosis over the right vulvar region and clitoris with increased extension to the left labia majora. On examination, the hematoma was more tense, tender, and newly warm to touch on the third day of admission.​ The skin integrity remained intact (Figure 2). The patient was booked and consented to an examination under general anesthetic as well as incision and evacuation of the hematoma.

A 3 cm incision was made at the point of maximum bulge of the hematoma at the mucocutaneous junction. The hematoma was evacuated, and interrupted sutures (2.0 Vicryl; Johnson & Johnson, New Brunswick, New Jersey) were used across bleeding vessels to achieve haemostasis. The cavity was irrigated with normal saline, and a ribbon gauze soaked in betadine was inserted into the cavity. Intravenous tranexamic acid was given intra-operatively and was continued for a total of 24 hours postoperatively. The total estimated blood loss was 150 ml. The following day after surgery, the ribbon gauze was removed along with the indwelling urethral catheter. The patient's pain was well controlled, she had no issues with micturition, and she was able to mobilize without difficulty. The patient was given oral amoxicillin/clavulanic acid for five days on discharge. The patient was advised not to insert anything per vagina for six weeks, including abstaining from sexual intercourse. She recovered well with no further complications or long-term adverse outcomes.

## Discussion

Large non-obstetric vulvar hematomas are uncommon gynecological emergencies, and there are only a few reported cases in the literature - a key contributing factor to the lack of consensus for the management of these cases. The majority of cases involve blunt trauma to the perineal region, which results in a hematoma due to the highly vascular anatomy of the vulvar region [1,4]. Non-obstetric vulvar hematomas secondary to consensual sexual intercourse, although rare, have been reported. Forceful sexual intercourse is an identified risk factor for developing such large and rapidly growing vulvar hematomas [6,7]. In the presented case, the patient had a history of repeated forceful fist placement in the vagina as part of consensual sexual intercourse, leading to her acute presentation. 

When a vulvar hematoma is identified, it is essential to determine if there is an extension and/or injury to the surrounding anatomy. This can be achieved with various imaging modalities such as transvaginal ultrasound, pelvic X-ray, computed tomography (CT) angiography, and magnetic resonance imaging (MRI) [2,8]. Evidence suggests that contrast-enhanced CT imaging is the recommended modality for assessing blunt trauma to the perineum as it can delineate injuries to the pelvic vasculature, genito-urinary tract, and pelvic floor musculoskeletal structures [8]. Although MRI also provides specific and detailed delineation for these cases, MRI is not readily accessible in some settings and provides a significantly higher cost compared to CT imaging [9]. 

There is no consensus in the literature regarding the best practice for the management of non-obstetric vulvar hematomas. Some limited studies suggest conservative management is appropriate for patients who are hemodynamically stable with acute hematomas that are not rapidly expanding [10]. Other limited studies found that conservative management was associated with prolonged hospital admission, increased risk of deterioration with need for antibiotics, blood transfusion, and subsequent need for surgical intervention [11, 12]. One small retrospective review [12] suggested that surgical intervention should be considered in cases of large vulvar hematomas (mean size of 9.5 cm), severe pain, distorted vulvar anatomy, and/or an obstructed urethral opening. In our presented case, the patient had a trial of conservative management for a large 10 cm vulvar hematoma with distorted vulvar anatomy and an almost obstructed urethral opening. The case was complicated by worsening signs and symptoms of pain and extension of the hematoma, which ultimately required surgical incision and evacuation of the hematoma. It is evident, particularly in the context of limited studies, that prompt surgical intervention would have reduced associated morbidity and duration of hospital admission in this case. 

There is a lack of data comparing the outcomes of conservative versus surgical management of non-obstetric vulvar hematoma, as well as identified risk factors for failure of conservative management. Future studies should evaluate conservative versus surgical management, with consideration to factors such as, but not limited to, size of the hematoma on presentation, effect on mobility, level of pain, extension to surrounding anatomy, as well as neurological and urological signs and symptoms. These further studies are necessary in order to establish evidence-based management guidelines for cases such as the one presented.

## Conclusions

In the presented case, the vulvar hematoma, although large, was stable and non-expanding on initial assessment. Therefore, conservative management was offered to and accepted by the patient, who then developed worsening pain and had extension of the hematoma over a 72-hour period despite conservative measures. She then required a surgical incision and evacuation of the hematoma. This case demonstrates the need for future studies in order to develop a consensus for when to pursue conservative versus surgical management in order to avoid morbidity and prolonged hospital admission.
